# Isolated Sixth Nerve Palsy as the First Manifestation of Cavernous Sinus Metastasis From Primary Breast Cancer

**DOI:** 10.7759/cureus.20094

**Published:** 2021-12-02

**Authors:** Anupam Singh, Pallavi Sharma, Himani Pal, Srishti Sharma, Aditi Dixit

**Affiliations:** 1 Ophthalmology, All India Institute of Medical Sciences, Rishikesh, IND; 2 Pathology, All India Institute of Medical Sciences, Rishikesh, IND

**Keywords:** metastatic breast cancer, brain metastasis, sixth nerve palsy, cavernous sinus metastasis, breast cancer

## Abstract

Cavernous sinus metastasis is a rare clinical finding, presenting most commonly with complaints of headache, diplopia, visual field defects, facial pain, and progressive neurological deficits. Many patients present with features of III, IV, and VI nerve palsies. We hereby report an unusual case of cavernous sinus metastasis from primary breast cancer in a 40-year-old female, who presented with binocular diplopia due to left VI nerve palsy as the first presenting complaint. The patient had a history of surgery for left breast cancer which was performed at another center. Contrast-enhanced computed tomography (CECT) scan of thorax and abdomen revealed a residual neoplastic left breast mass with satellite nodules, left axillary lymphadenopathy, and hepatic, splenic, and skeletal metastasis. Contrast-enhanced magnetic resonance imaging (CE-MRI) of brain and orbit showed enhancing lesion of 20 mm x 10 mm along the lateral wall of left cavernous sinus and left petrous apex. She was referred to radiation oncology department for further management. This case report highlights the importance of ophthalmologists in such life-threatening conditions, who may first present to them.

## Introduction

The risk of symptomatic central nervous system (CNS) metastasis among patients from breast cancer ranges from 10% to 16% among living patients; however, autopsy studies have reported the risk up to 30% [1]. The cerebellum (33%) is the most common site of CNS metastasis followed by the frontal lobe (26%) in these patients. Headache (35%) is the most common presenting symptom followed by vomiting (26%), nausea (23%), hemiparesis (22%), visual changes (13%), and seizures (12%) [2].

Breast cancer metastasis presenting as cranial nerve dysfunctions occur mainly due to the bony metastases at the base of the skull [3], which either compresses or inﬁltrates the nerve. But clinical presentation with cranial nerve dysfunction without skull base metastasis should raise the suspicion of either the cavernous sinus or orbital apex involvement [2].

Cavernous sinus metastases are often associated with headache, facial pain, diplopia, visual field defects, or clinical features of III, IV, and VI cranial nerves' involvement [4]. The involvement of the cavernous sinus from breast cancer has been reported by only a few authors [5,6], probably due to its infrequent presentation and misdiagnosed nature.

We hereby report a rare case of breast cancer, who presented with binocular diplopia as the first clinical presentation of cavernous sinus metastasis due to primary breast cancer.

## Case presentation

**Figure 1:**
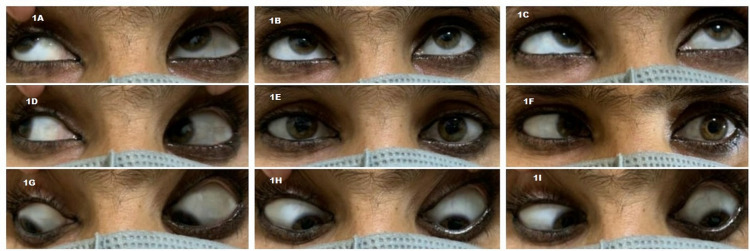
Clinical photographs showing nine gazes (1A-1I). There is orthophoria in primary position (1E) with limitation of abduction in left eye (1C, 1F, 1I), suggestive of left abducens nerve palsy.

A 40-year-old female presented with complaints of double vision for two months. She was diagnosed with invasive ductal carcinoma of the left breast (grade II) and underwent left breast lumpectomy four months back at another center. The patient did not give a history of any neoadjuvant therapy and immunohistochemistry was not done after the lumpectomy. There was no history of hypertension, diabetes mellitus, tuberculosis, or trauma. On examination, the best-corrected visual acuity was 20/20 in both eyes. Intraocular pressure with non-contact tonometer, pupillary reflexes, and anterior segment was normal in both eyes. On Hirschberg test, she was orthophoric in primary gaze. Extraocular motility evaluation revealed limited abduction of the left eye. Ocular movements in the rest of all other directions were full and free (Figure 1A-1I).

On prism bar cover test (PBCT) with right eye fixing, there was 15 prism diopter (PD) of left esotropia on near fixation, which increased to 25 PD on distant fixation. On PBCT with left eye fixing, there was a secondary deviation of 25 PD at near and 40 PD at distant fixation. Diplopia charting with red-green glasses revealed horizontal uncrossed diplopia increasing in left gaze and at distance fixation. Posterior segment examination with indirect ophthalmoscope was unremarkable in both eyes. Corneal sensation, colour vision, contrast sensitivity, and visual fields were within normal limits in both eyes.

**Figure 2:**
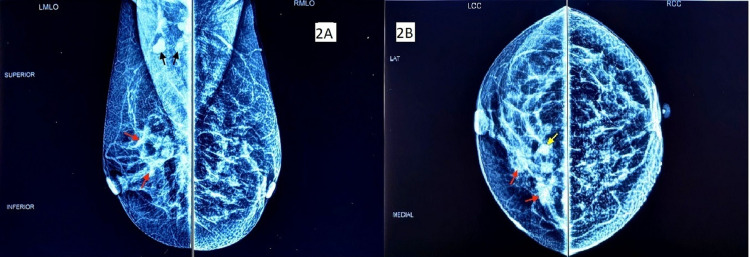
Mammography of both breasts (after left breast lumpectomy). 2A. Mediolateral oblique view showing an asymmetrical area of increased density in the upper quadrant of the left breast (red arrows). Few enlarged lymph nodes are also seen in the left axilla (black arrows). 2B. Craniocaudal view showing a rounded, well-circumscribed focal increased density in the inner upper quadrant of the left breast (yellow arrow) along with asymmetrical areas of increased density (red arrows).

Based on the above clinical findings a provisional diagnosis of left acquired sixth nerve palsy was made. As the patient had a history of primary breast malignancy, she was referred to the breast clinic of the institute for re-evaluation where she was suspected of having a residual lesion in the left breast. She was advised to undergo mammography, which confirmed the presence of a residual neoplastic lesion in the left breast (Figure 2A, 2B).

**Figure 3:**
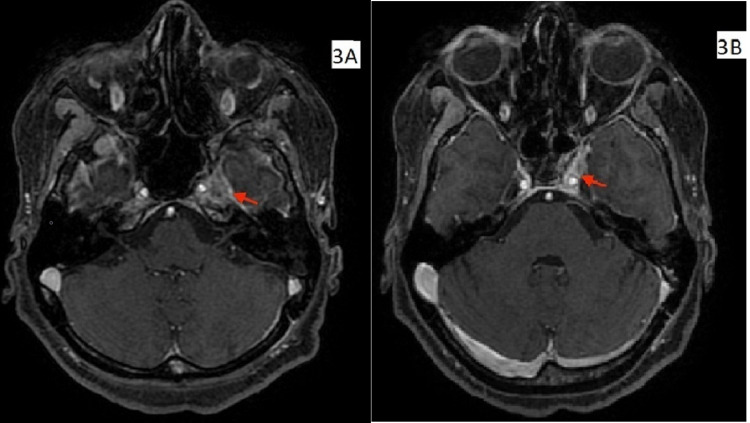
3A, 3B. Post-contrast T1-weighted axial images of contrast-enhanced magnetic resonance imaging of brain showing enhancing lesion along lateral wall of left cavernous sinus and left petrous apex, also involving left foramen ovale and left mandibular nerve with diffuse enhancing dural thickening and multifocal enhancing plaque-like dural thickening up to 4 mm along left cerebral convexity in insular region (red arrows).

Contrast-enhanced computed tomography (CECT) scan of thorax and abdomen showed a neoplastic left breast mass with satellite nodules, left axillary lymphadenopathy, and hepatic, splenic, and skeletal metastasis. Contrast-enhanced magnetic resonance imaging (CE-MRI) of brain and orbit showed enhancing lesion of 20 mm x 10 mm along the lateral wall of the left cavernous sinus and left petrous apex, also involving left foramen ovale and left mandibular nerve (Figure 3A, 3B, red arrows) with diffuse enhancing dural thickening and multifocal enhancing plaque-like dural thickening up to 4 mm along the left cerebral convexity in the insular region.

**Figure 4:**
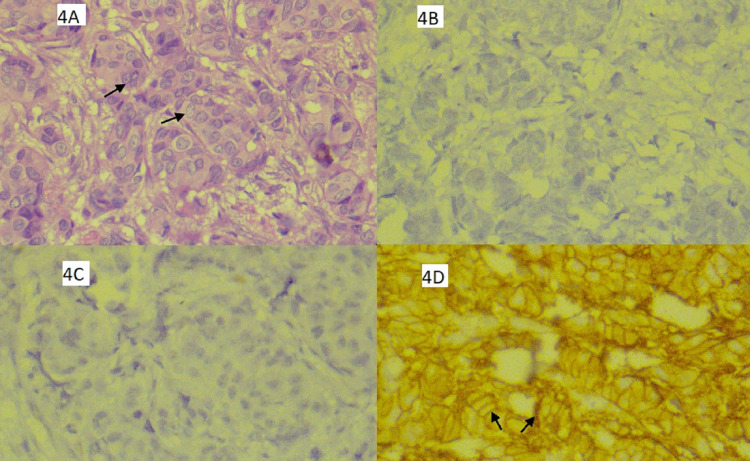
Histopathological findings. 4A. Nottingham Grade III invasive ductal carcinoma: Sheets of individual and nests of cells showing poorly differentiated tubules with marked nuclear pleomorphism and high mitotic activity (black arrows). 4B. Weak staining of tissues, showing oestrogen receptor negative status. 4C. Weak staining of tissues, showing progesterone receptor negative status. 4D. Strong complete brownish membrane staining, showing human epidermal growth factor receptor 2 positive status (black arrows).

Histopathology of the left breast lump revealed invasive ductal carcinoma - Nottingham Grade III (Figure 4A) - and immunohistochemistry study suggested tumour as oestrogen receptor (ER)/ progesterone receptor (PR) negative (Figure 4B, 4C, respectively) and human epidermal growth factor receptor 2 neu (HER 2 neu) positive (Figure 4D).

Therefore, the final diagnosis of acquired sixth nerve palsy due to cavernous sinus metastasis from residual primary breast malignancy (stage T4bN1M1) was made. The patient was referred to radiation oncology and was advised radiotherapy of 30 grays in 10 fractions.

## Discussion

Carcinoma of the breast is the second most common cause of secondaries to the brain after lung carcinoma [2]. Brain may be the first site of metastasis in 12% of patients with breast malignancy [7]. In the past few years there has been rising trend in incidence of brain metastasis due to breast cancer, most probably due to advances in neuroimaging leading to increased detection of brain metastasis and novel therapies for breast cancer leading to longer patient survival [8,9]. According to the literature, brain metastases from breast cancer (BMBC) occur more frequently among younger women, those with larger tumours or higher nuclear grade, in certain subtypes such as ER-negative, triple-negative, HER2-overexpressing tumours, and those with nodal metastases [1]. The overall prognosis of patients with BMBC has improved in recent years and it is now more favorable than that of patients with brain metastasis from lung cancer [2,10].

The patient may also present with cranial nerve palsies if there is involvement of brainstem, cavernous sinus, or orbital apex by tumour or metastasis. Metastases to cavernous sinus occur in less than 1% of patients with cancer, and it is considered a late event when patients have disseminated disease. The cavernous sinus contains vital neurovascular structures that may be affected by vascular, neoplastic, infective, and infiltrative lesions arising in the cavernous sinus proper or via extension from adjacent intra- and extracranial regions [6,10].

Patients with cavernous sinus syndrome usually present with paresis of one or more cranial nerves (III−VI), which may be associated with painful ophthalmoplegia. Sixth nerve is the most commonly involved nerve as it has the longest intracranial course between the brainstem and the eye, which makes it vulnerable to be affected at multiple levels, and it is the nerve that passes through the substance of cavernous sinus. The abducens nerve begins in the pons near the seventh cranial nerve before exiting the brainstem. At this point, it travels into the subarachnoid space and moves along the skull at the clivus. It then travels to the basal skull at the petrous apex of the temporal bone, where it enters the cavernous sinus. The abducens nerve then enters the orbit via the superior orbital fissure and innervates the lateral rectus muscle, which is responsible for abduction of the eye [11]. Abducens nerve palsy leads to lateral rectus palsy, resulting in an inability to abduct the eye and horizontal diplopia. In our patient, she had pre-existing malignancy, breast carcinoma, complaining of horizontal diplopia due to limitation of abduction corresponding to left abducens nerve palsy. In the post-contrast T1-weighted CE-MRI, enhancing lesion of 20 mm x 10 mm was seen along the lateral wall of the left cavernous sinus and left petrous apex, which was suggested to be metastasized from primary breast carcinoma. Sixth nerve was likely to be involved while passing through petrous apex of temporal bone or through the substance of cavernous sinus. The patient was referred to the radiation oncology department where she was advised to receive radiotherapy and she was receiving cycles of radiotherapy at the time of writing this case report. Although the prognosis for advanced carcinoma of the breast with metastasis is poor (bone metastasis being the best and brain metastasis being the worst), the currently available chemotherapeutic and radiation therapies offer prolongation of survival rates [12,13].

Isolated abducens nerve palsy in a patient with primary breast malignancy without any history of trauma, hypertension, and diabetes mellitus raised the suspicion of brain metastasis. Further, young age (40 years), ER-negative, and HER 2 neu-positive primary breast malignancy with nodal metastasis were the high-risk factors for brain metastasis. Diplopia was the first and only presenting feature of the metastatic breast malignancy, which led the patient to consult an ophthalmologist. Prompt diagnosis and multidisciplinary approach in such cases can be both life- and sight-saving.

## Conclusions

Cavernous sinus metastasis due to breast malignancy is rare but one of the life-threatening conditions. The most common presenting complaints are headache, facial pain, and diplopia due to III, IV, or VI nerve palsy. These patients may first present to an ophthalmologist. Therefore, ophthalmologists should be aware of the possibility of such secondaries as underlying cause of headache and diplopia. Thus, they can play a key role in such cases by prompt diagnosis and multidisciplinary approach can be life-saving.
